# Identification of potential ferroptosis-related biomarkers and a pharmacological compound in diabetic retinopathy based on machine learning and molecular docking

**DOI:** 10.3389/fendo.2022.988506

**Published:** 2022-11-24

**Authors:** Jingying Liu, Xiaozhuang Li, Yanhua Cheng, Kangcheng Liu, Hua Zou, Zhipeng You

**Affiliations:** Jiangxi Province Division of National Clinical Research Center for Ocular Diseases, Jiangxi Clinical Research Center for Ophthalmic Disease, Jiangxi Research Institute of Ophthalmology and Visual Science, Affiliated Eye Hospital of Nanchang University, Nanchang, Jiangxi, China

**Keywords:** diabetic retinopathy, ferroptosis, biomarkers, glutathione, *in silico*

## Abstract

**Background:**

Diabetic retinopathy (DR), a neurovascular disease, is a leading cause of visual loss worldwide and severely affects quality of life. Several studies have shown that ferroptosis plays an important role in the pathogenesis of DR; however, its molecule mechanism remains incompletely elucidated. Hence, this study aimed to investigate the pathogenesis of ferroptosis and explore potential ferroptosis-related gene biomarkers and a pharmacological compound for treating DR.

**Methods:**

Ferroptosis-related differentially expressed genes (DEGs) were identified in the GSE102485 dataset. Functional enrichment analyses were then performed and a protein-protein interaction (PPI) network was constructed to screen candidates of ferroptosis-related hub genes (FRHGs). FRHGs were further screened based on least absolute shrinkage and selection operator (LASSO) regression and random forest algorithms, and were then validated with the GSE60436 dataset and previous studies. A receiver operating characteristic (ROC) curve monofactor analysis was conducted to evaluate the diagnostic performance of the FRHGs, and immune infiltration analysis was performed. Moreover, the pharmacological compound targeting the FRHGs were verified by molecular docking. Finally, the FRHGs were validated using quantitative real-time polymerase chain reaction (qRT-PCR) analysis.

**Results:**

The 40 ferroptosis-related DEGs were extracted, and functional enrichment analyses mainly implicated apoptotic signaling, response to oxidative stress, ferroptosis, and lipid and atherosclerosis pathways. By integrating the PPI, LASSO regression, and random forest analyses to screen the FRHGs, and through validation, we identified five FRHGs that performed well in the diagnosis (*CAV1*, *CD44*, *NOX4*, *TLR4*, and *TP53*). Immune infiltration analysis revealed that immune microenvironment changes in DR patients may be related to these five FRHGs. Molecular docking also showed that glutathione strongly bound the CAV1 and TLR4 proteins. Finally, the upregulated expression of FRHGs (*CD44*, *NOX4*, *TLR4*, and *TP53*) was validated by qRT-PCR analysis in human retinal capillary endothelial cells cultured under high-glucose environment.

**Conclusions:**

*CAV1, CD44, NOX4, TLR4*, and *TP53* are potential biomarkers for DR and may be involved in its occurrence and progression by regulating ferroptosis and the immune microenvironment. Further, glutathione exhibits potential therapeutic efficacy on DR by targeting ferroptosis. Our study provides new insights into the ferroptosis-related pathogenesis of DR, as well as its diagnosis and treatment.

## 1 Introduction

Diabetic retinopathy (DR), a specific neurovascular complication of both type 1 and 2 diabetes, is among the leading causes of visual impairment and blindness in adults worldwide, with an estimated 191 million affected patients by 2030 (1, 2). It is generally acknowledged that the prevalence of DR increases with the duration of diabetes. For type 1 diabetes, approximately 25%, 60%, and 80% of patients will develop DR after 5, 10, and 15 years, respectively. In less than 5 years, the incidence of DR in patients with type 2 diabetes is 40% and 24% for those who do and do not take insulin, respectively. After 19 years, these rates rise to 84% and 53%, respectively (3). The occurrence and progression of DR are so latent that detection is difficult. When visual impairment does occur, the optimal time for diagnosis and therapy has usually passed (4). Thus, it is imperative to further investigate the pathogenesis of DR, distinguish novel biomarkers for diagnosis, and identify pharmacological compounds for targeted treatment.

Ferroptosis is a recently identified type of cell death whose main characteristics are iron-dependent accretion of lipid reactive oxygen species and inhibition of the cystine/glutamate antiporter system Xc^−^, leading to decreased cystine uptake and glutathione (GSH) synthesis (5). Ferroptosis may fatally damage cells and result in certain eye diseases, such as glaucoma, retinal ischemia-reperfusion injury, and age-related macular degeneration (6, 7). Recent evidence has revealed the role of ferroptosis in DR. Damage to retinal pigment epithelial (RPE) cells, the resulting destruction to the blood-retina barrier, and increased permeability of human retinal capillary endothelial cells (HRCECs) are key features in the occurrence and progression of DR. It has been reported that ferroptosis serves as a cell death pathway for RPE cells and HRCECs in DR (8, 9). Nevertheless, the DR-related pathologic mechanisms, signaling pathways, and gene biomarkers in ferroptosis have not yet been clarified.

GSH, a bioactive substance involved in cellular metabolism and antioxidant defense, is utilized by glutathione peroxidase 4 (GPX4) to eliminate phospholipid peroxides and protect cells from ferroptosis (10). Studies have shown that enhancement of intracellular GSH activity by natural compounds can alleviate DR by modulating inflammation, oxidative stress, endoplasmic reticulum stress, and autophagy (11). Nevertheless, the pharmacological activity of GSH to target ferroptosis in DR remains unclear.

In this study, we collected RNA-sequencing dataset from the Gene Expression Omnibus (GEO) database and downloaded ferroptosis-related genes from the FerrDb database. We first identified ferroptosis-related differentially expressed genes (DEGs) and performed functional enrichment analyses. Protein-protein interaction (PPI), least absolute shrinkage and selection operator (LASSO) regression, and random forest analyses were further utilized to identify ferroptosis-related hub genes (FRHGs), and another GEO dataset and previous studies were utilized for validation. In addition, we used CIBERSORT to analyze the immune microenvironment in DR. Then, molecular docking between GSH and the FRHG-encoded proteins was performed to validate their prospective application in DR treatment. Finally, the FRHGs were validated using quantitative real-time polymerase chain reaction (qRT-PCR) analysis in the *in vitro* DR model. Our study provides new insights into the potential pathogenesis associated with ferroptosis at the molecular level, novel diagnostic biomarkers, and a pharmacological compound targeting ferroptosis in DR. This is expected to provide valuable information in the future for the accurate diagnosis of DR, as well as drug discovery and development.

## 2 Materials and methods

### 2.1 Data collection, preprocessing, and quality control

**Figure 1:**
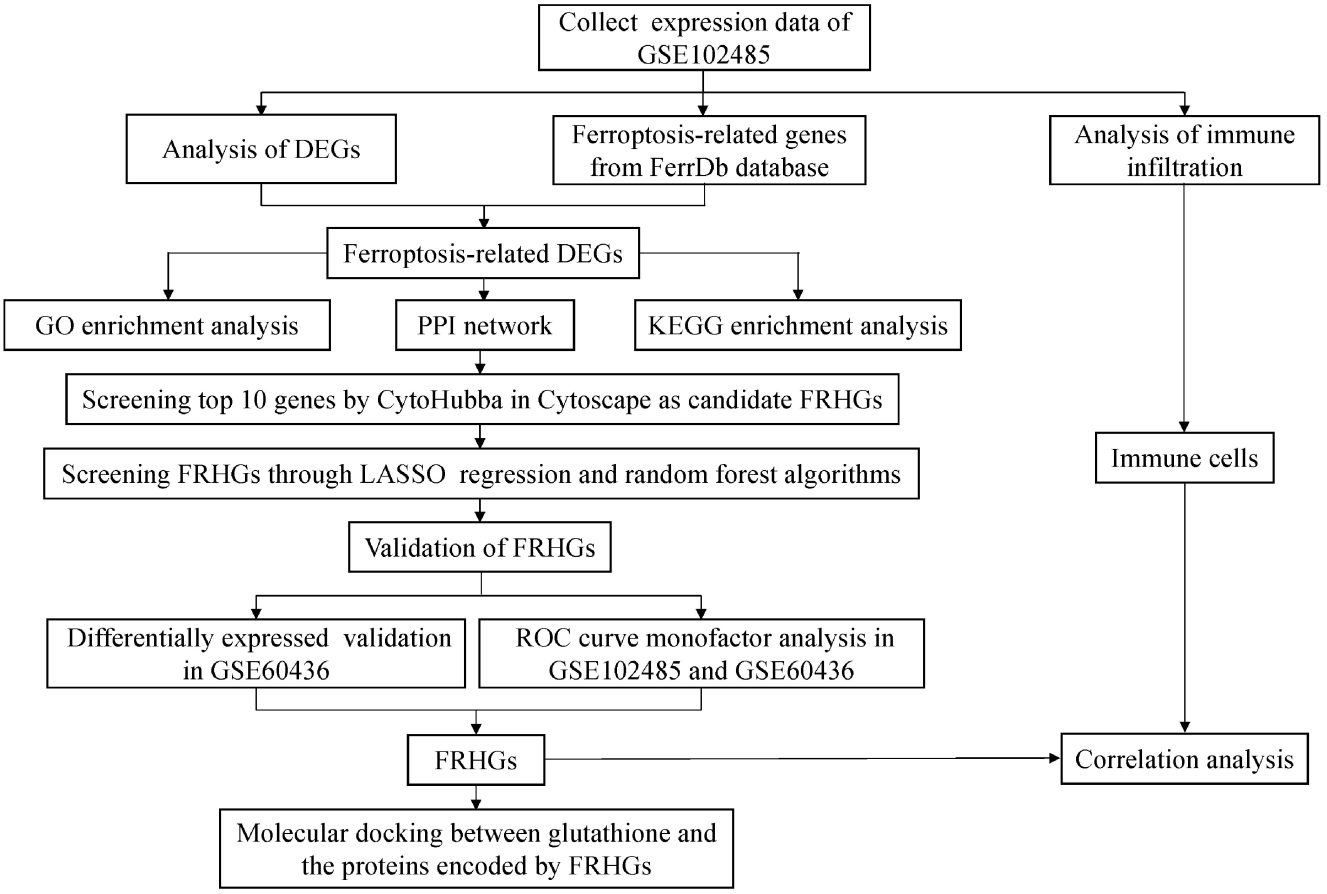
Workflow of data analyses utilized in this study. DEGs, differentially expressed genes; GO, Gene Ontology; PPI, protein-protein interaction; KEGG, Kyoto Encyclopedia of Genes and Genomes; FRHGs, ferroptosis-related hub genes; LASSO, least absolute shrinkage and selection operator; ROC, receiver operator characteristic.

The two DR datasets used in this study were downloaded from the GEO database (https://www.ncbi.nlm.nih.gov/gds). The first transcriptome dataset was the test dataset (GSE102485) and the second microarray dataset was the validation dataset (GSE60436); both are shown in **Table 1**. Two hundred and fifty-nine ferroptosis-related genes were downloaded from the FerrDb database (http://www.zhounan.org/ferrdb/) (12). The workflow of this study is shown in **Figure 1**. The GSE102485 dataset also contained non-DR samples, which were excluded. Thus, our study only utilized DR and normal samples for the downstream bioinformatic analyses.

**Table 1:** Diabetic retinopathy (DR) datasets from the GEO database.

Dataset ID	Platform	DR	Normal	Other retinopathy
GSE102485	GPL18573	22	3	5
GSE60436	GPL6884	6	3	0

Data processing was performed using R (version 4.1.1) as follows. First, we transformed the Ensembl IDs to gene symbols, and protein-coding genes in the GSE102485 dataset were selected for analyses. Second, we performed ID conversion in the GSE60436 dataset. Third, the average expression value was regarded as the gene expression value when multiple Ensembl IDs/probes corresponded to the same gene symbol.

The DESeq2 package (13) was utilized to normalize the raw count data of mRNAs for further principal component analysis (PCA). The FactoMineR package (14) for dimensionality reduction was used for PCA to evaluate the data quality.

### 2.2 Identification of DEGs and ferroptosis-related DEGs

We analyzed gene expression of the GSE102485 dataset *via* the DESeq2 package to identify DEGs. As suggested by the DESeq2 package tutorial, genes with low read counts were not worthy of further analyses. Hence, mRNAs with a mean count less than one and median count equal to zero were excluded in our study. Differential expression analysis then was performed in which the normalization processes of count data were incorporated into the DESeq2 workflow. The false discovery rate was calculated by the Benjamini–Hochberg method and applied to correct the statistical significance of multiple testing (15). The DEGs were screened based on a threshold of |log2 fold-change (FC)| ≥ 2 and false discovery rate < 0.05. Finally, we constructed a volcano plot using the ggplot2 package (16) to visualize the results.

We intersected the 259 ferroptosis-related genes with the DEGs to identify ferroptosis-related DEGs. The online analysis tool Venny2.1 (https://bioinfogp.cnb.csic.es/tools/venny/index.html) was utilized to construct a venn diagram to visualize the results. Then, the pheatmap package was utilized to construct a heat map to visualize expression of the ferroptosis-related DEGs.

### 2.3 Functional enrichment analyses of ferroptosis-related DEGs

The clusterProfiler (17) and GOplot (18) packages were utilized to perform Gene Ontology (GO) and Kyoto Encyclopedia of Genes and Genomes (KEGG) enrichment analyses for the ferroptosis-related DEGs. A bar plot was used to show the top 30 GO terms, including biological process, cellular component, and molecular function, and a chord plot was used to show crosstalk between the ferroptosis-related DEGs and top five GO terms, linking them by ribbons. A circle plot was used to show the top 10 KEGG pathways. A *p*-value < 0.05 was considered statistically significant.

### 2.4 PPI network construction and screening of FRHGs

The STRING database (19) was utilized to observe interactions between the ferroptosis-related DEGs. Cytoscape software (version 3.7.2) was used to construct and visualize the PPI network. Candidate FRHGs were then identified using the MCC algorithm of the Cytoscape plug-in CytoHubba. The 10 genes with the highest scores were screened as candidate FRHGs and displayed in the Cytoscape software.

LASSO regression, a machine learning algorithm with dual characteristics of subset selection and ridge regression, is widely utilized to screen the best variables by finding the lambda value when the classification model error is the least (20). The glmnet package (21) was used to perform LASSO regression analysis. Expression of the 10 candidate FRHGs was analyzed using LASSO regression with a binomial model and lambda value equal to the minimum mean cross-validated error to screen most likely FRHGs. Random forest, another machine learning algorithm for training and predicting samples with high accuracy based on constructing a multitude of decision trees, is widely utilized to identify and verify potential predictors (22). Thus, the random forest algorithm was utilized to verify the reliability of the LASSO regression analysis using the randomForest package (23). The out-of-bag error was calculated to evaluate the classification performance of the combined FRHGs identified by LASSO regression. The mean decrease accuracy (MDA) and mean decrease Gini (MDG) were positively correlated with the importance of each variable. Therefore, these FRHGs were sorted by MDA and MDG indexes.

### 2.5 Dataset validation of FRHGs

First, the normalized expression values of the FRHGs in the GSE60436 dataset were extracted and groups were compared utilizing *t*-tests; *p*-values < 0.05 were considered statistically significant. We utilized the ggpubr package to visualize these results. Second, receiver operating characteristic (ROC) curve monofactor analysis was performed on the GSE102485 and GSE60436 datasets to confirm these FRHGs. The ROC curve was visualized using Hiplot software (https://hiplot.com.cn/). Any gene with an area under the ROC curve > 0.9 was considered to have great diagnostic value.

### 2.6 Immune infiltration analyses

The CIBERSORT package (24) was utilized to analyze immune cell infiltration in DR and normal samples. The normalized gene expression data was transformed into immune cell information by the CIBERSORT deconvolution algorithm. Linear regression analysis was used to analyze the correlation between FRHGs expression and immune cells. A *p*-value < 0.05 was considered statistically significant. The results were visualized using the ggplot2 package.

### 2.7 Molecular docking

The 2D chemical structure of GSH was downloaded from PubChem. Various databases were utilized to identify whether the FRHGs were potential targets of GSH, as previously described (25). Molecular docking was utilized to simulate intermolecular binding patterns between GSH and the target proteins. The protein structures of identified GSH targets were obtained from the PDB database (https://www.rcsb.org/). Then, MGLTools (version 1.5.7) in AutoDock (26) was utilized to conduct the docking analysis. After converting the pdbqt format to pdb using OpenBabel, PyMOL was then used to visualize the molecular docking results. The docking parameter setting was assessed according to the binding energy of the ligand.

### 2.8 External validation of GPX4, SLC7A11 and FRHGs

#### 2.8.1 Cell culture and cell grouping

Human retinal capillary endothelial cells (HRCECs) were cultured in low-glucose DMEM (Solarbio, China) containing 10% foetal bovine serum (Biological Industries, Israel) at 37°C with 5% carbon dioxide. HRCECs cultured in the medium containing 5.5 mmol/L glucose were used as the normal control group (NG group) and cultured in the medium containing 30 mmol/L glucose were used as the high-glucose group (HG group). Mannitol was used as the control to eliminate the influence of osmotic pressure, namely, the MA group. The model cells under HG group were cultured with high-glucose DMEM for 12, 24, and 48 h.

#### 2.8.2 Western blot analysis

Total cellular protein was extracted using Radio Immunoprecipitation Assay (RIPA) Lysis Buffer (Solarbio, China). The proteins were denatured and then separated using 10% SDS-PAGE and then transferred to polyvinylidene fluoride membranes. Next, these membranes were blocked with 5% non-fat milk at room temperature for 2 h and incubated with primary antibodies against GPX4 (ab125066, Abcam) and SLC7A11 (ab175186, Abcam) at 4°C overnight. Subsequently, the membranes were incubated with secondary antibodies conjugated to horseradish peroxidase (BA1054, BOSTER) at room temperature for 1 h. The ECL developer (US EVERBRIGHT, China) was added to the membranes and Imaging System (SYNGENE, Britain) was used to visualize the immunoreactive protein bands. β-actin (AC026, ABclonal) was used as the internal reference.

#### 2.8.3 Quantitative real-time polymerase chain reaction (qRT-PCR) analysis

Total cellular RNA was isolated using RNA Extraction reagent (Servicebio, China) according to manufacturer’s instructions. Total RNA was then reverse transcribed to cDNA using SweScript RT I First Strand cDNA Synthesis Kit (Servicebio, China), and RT-PCR was performed using 2 × SYBR Green qPCR Master Mix (None ROX) (Servicebio, China). The primer sequences used in this study were shown in **Supplementary Table 1**. Relative change in gene expression was calculated with the 2^−ΔΔCt^ method using GAPDH as the internal reference.

### 2.9 Statistical analysis

All the experimental data, taken from at least three independent experiments, was statistically analyzed using GraphPad Prism software (version 8.0.1). Significance levels were determined by the unpaired Student’s t-test between the two groups or the one-way analysis of variance (ANOVA) among multiple groups. A *p*-value < 0.05 was considered statistically significant.

## 3 Results

### 3.1 Data collection, preprocessing, and quality control

After expression data collection and preprocessing, all samples were assessed by PCA (**Supplementary Figure 1**). The results showed that the DR samples were distinctly different from the normal samples, which confirmed the repeatability of the GSE102485 data and suitability for downstream analysis.

### 3.2 Identification of DEGs and ferroptosis-related DEGs

**Figure 2:**
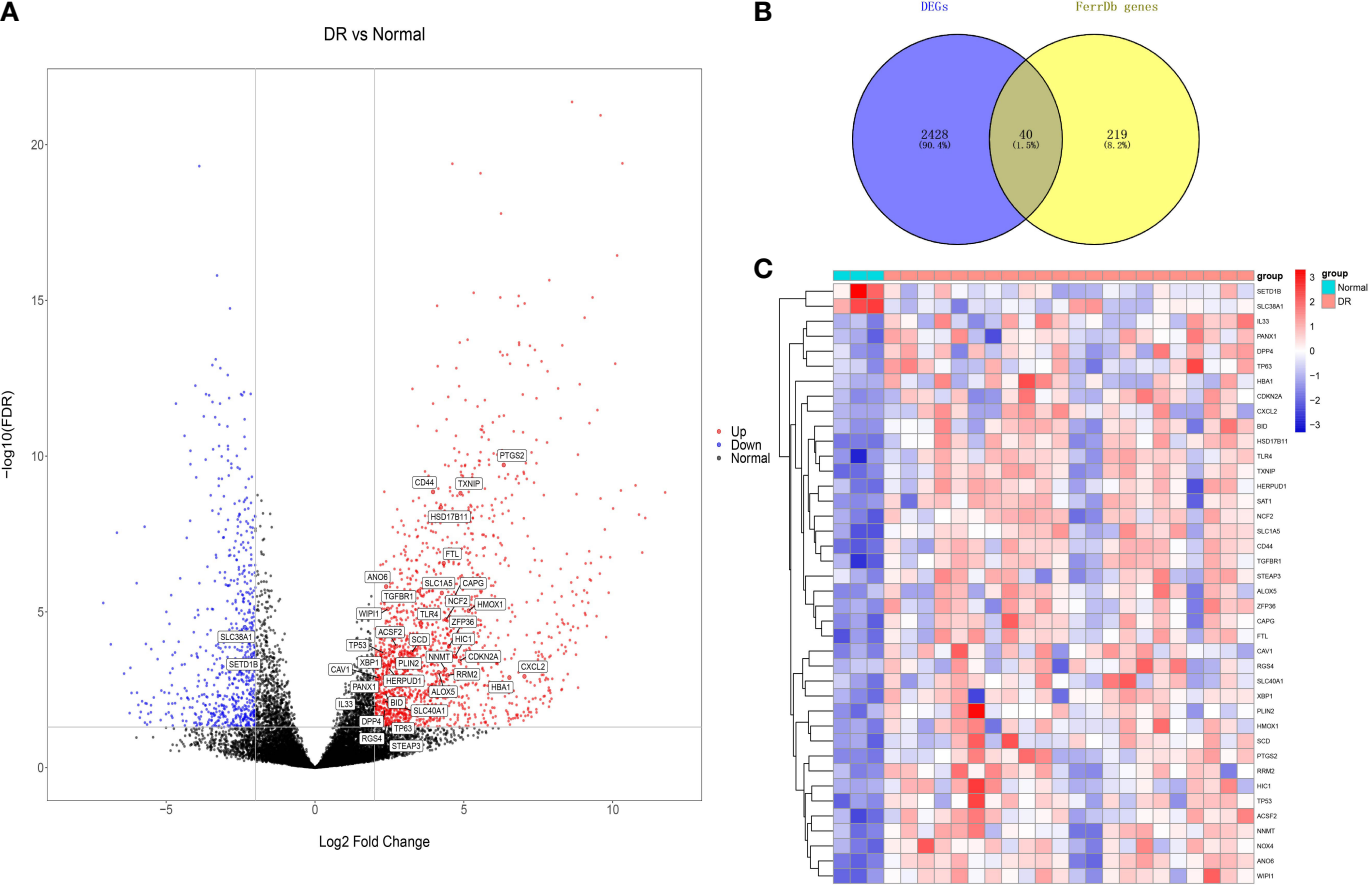
Differentially expressed genes (DEGs) analysis. **(A)** Volcano plot of DEGs. **(B)** Venn diagram of ferroptosis-related DEGs, and their gene symbols were shown in **(A)**. **(C)** Heat map of ferroptosis-related DEGs. DR, diabetic retinopathy.

According to the assigned threshold, 2468 DEGs were detected, of which 1861 were upregulated and 652 were downregulated in DR (**Figure 2A**). We then intersected 259 ferroptosis-related genes with the DEGs, identifying 40 ferroptosis-related DEGs (**Figure 2B**), of which 38 were upregulated and 2 were downregulated in DR. The gene symbols of ferroptosis-related DEGs are shown in **Figure 2A**. A heat map of these ferroptosis-related DEGs revealed variations in relative gene expression among the DR and normal samples (**Figure 2C**).

### 3.3 Functional enrichment analyses of ferroptosis-related DEGs

**Figure 3:**
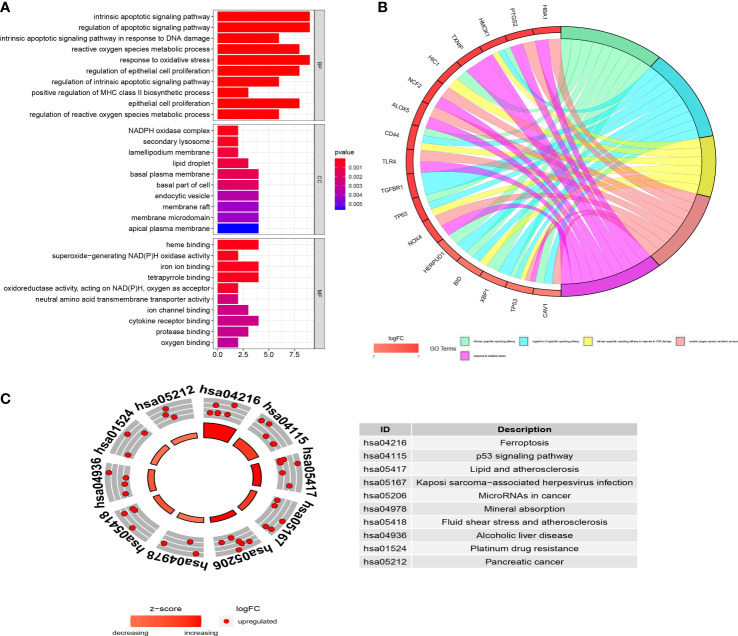
The functional enrichment analyses of the ferroptosis-related differentially expressed genes (DEGs). **(A)** GO analysis of the ferroptosis-related DEGs. **(B)** Chord plot shows the distribution of ferroptosis-related DEGs in different GO terms. Symbols of ferroptosis-related DEGs are shown on the left side of the plot with their logFC values indicated by color scale. **(C)** KEGG analysis of the ferroptosis-related DEGs. The inner ring is a bar plot where height displays the significance of the term, and the outer ring displays scatter plots which indicate the expression levels (logFC) for the genes in each term. BP, biological process; CC, cellular component; MF, molecular function; logFC, log2 fold-change.

In GO enrichment analysis, ferroptosis-related DEGs were significantly enriched in the intrinsic apoptotic signaling pathway, regulation of apoptotic signaling pathway, intrinsic apoptotic signaling pathway in response to DNA damage, reactive oxygen species metabolic process, and response to oxidative stress under the biological process term; NADPH oxidase complex, secondary lysosome, and lamellipodium membrane under the cellular component term; and heme binding, superoxide-generating NAD(P)H oxidase activity, and iron ion binding under the molecular function term (**Figure 3A**). The results of crosstalk analyses of genes and GO terms revealed that the functions of ferroptosis-related DEGs in DR might be the result of mutual relationships among multiple gene functions, which are shown in **Figure 3B**. In KEGG enrichment analysis, the ferroptosis-related DEGs were significantly enriched in ferroptosis, the p53 signaling pathway, and lipid and atherosclerosis pathways (**Figure 3C**).

### 3.4 PPI network construction and screening of FRHGs

**Figure 4:**
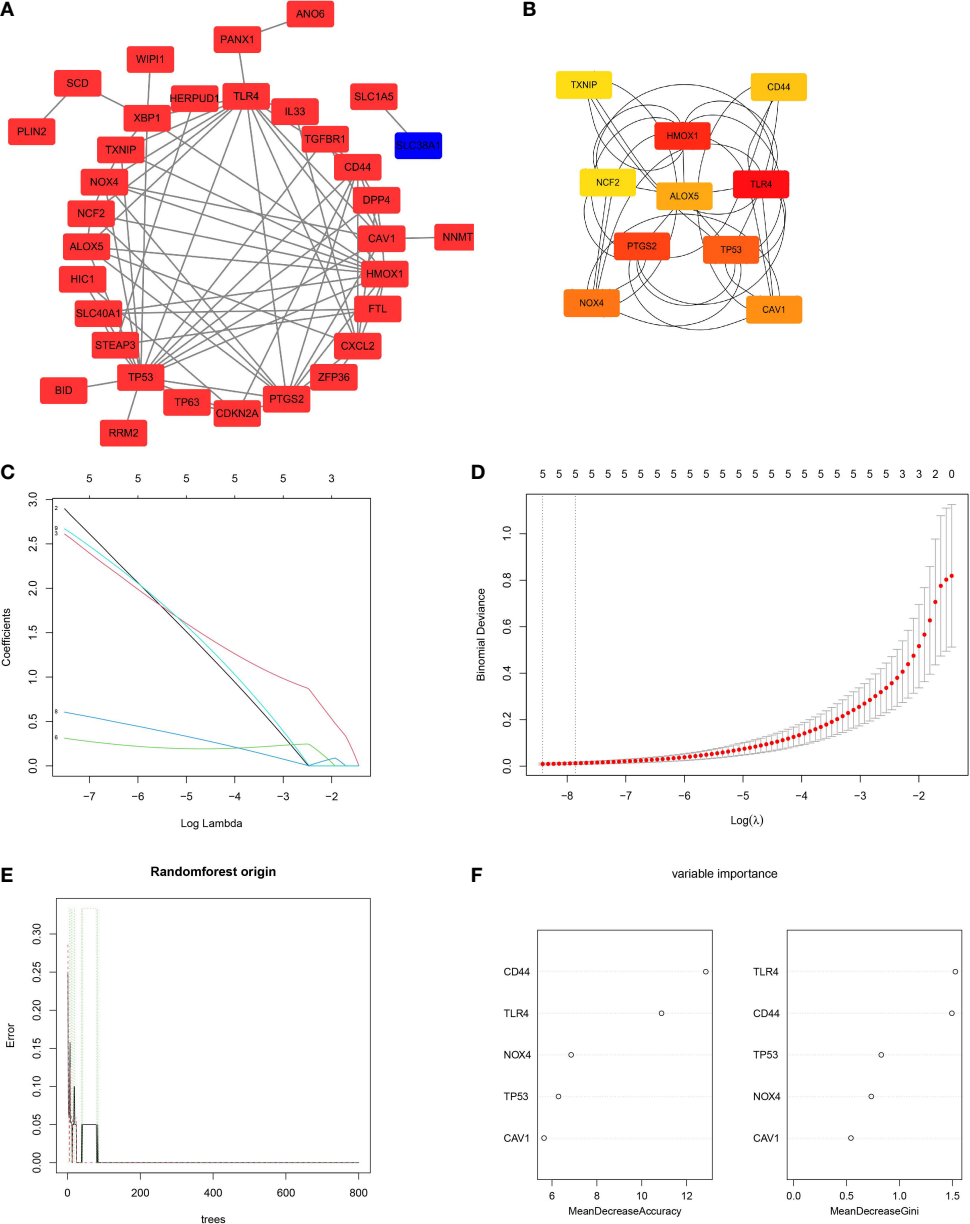
Protein-protein interaction (PPI) analysis and screening of ferroptosis-related hub genes (FRHGs). **(A)** The PPI network of ferroptosis-related DEGs. Red rectangles represent upregulated genes, while the blue rectangle represents a downregulated gene. **(B)** Network of the candidate FRHGs. A redder color represents a higher score in Cytoscape based on the MCC algorithm. **(C, D)** Least absolute shrinkage and selection operator (LASSO) regression algorithm to screen five FRHGs. **(E, F)** Construction and evaluation of random forest model based on the five FRHGs screened by LASSO regression. **(E)** Trend of the model errors based on the number of decision trees. **(F)** The importance of all variables in the random forest model.

According to the PPI network results, there were interactions between the identified ferroptosis-related DEGs (**Figure 4A**). The genes with the top 10 scores were screened as candidate FRHGs, namely, *TXNIP*, *CD44*, *HMOX1*, *NCF2*, *ALOX5*, *TLR4*, *PTGS2*, *TP53*, *NOX4*, and *CAV1* (**Figure 4B**).

To identify the best FRHGs, LASSO regression was used to analyze the 10 candidate FRHGs. Five genes, *CAV1*, *CD44*, *NOX4*, *TLR4*, and *TP53*, were identified (**Figures 4C, D**). The random forest algorithm was then used to efficiently predict the combined classification performance of these five genes and evaluate the importance of each gene. The out-of-bag error of the random forest model was 0%, and the five genes were ranked by MDA and MDG indexes (**Figures 4E, F**). The results showed high reliability and important metrics for the five FRHGs.

### 3.5 Dataset validation of FRHGs

**Figure 5:**
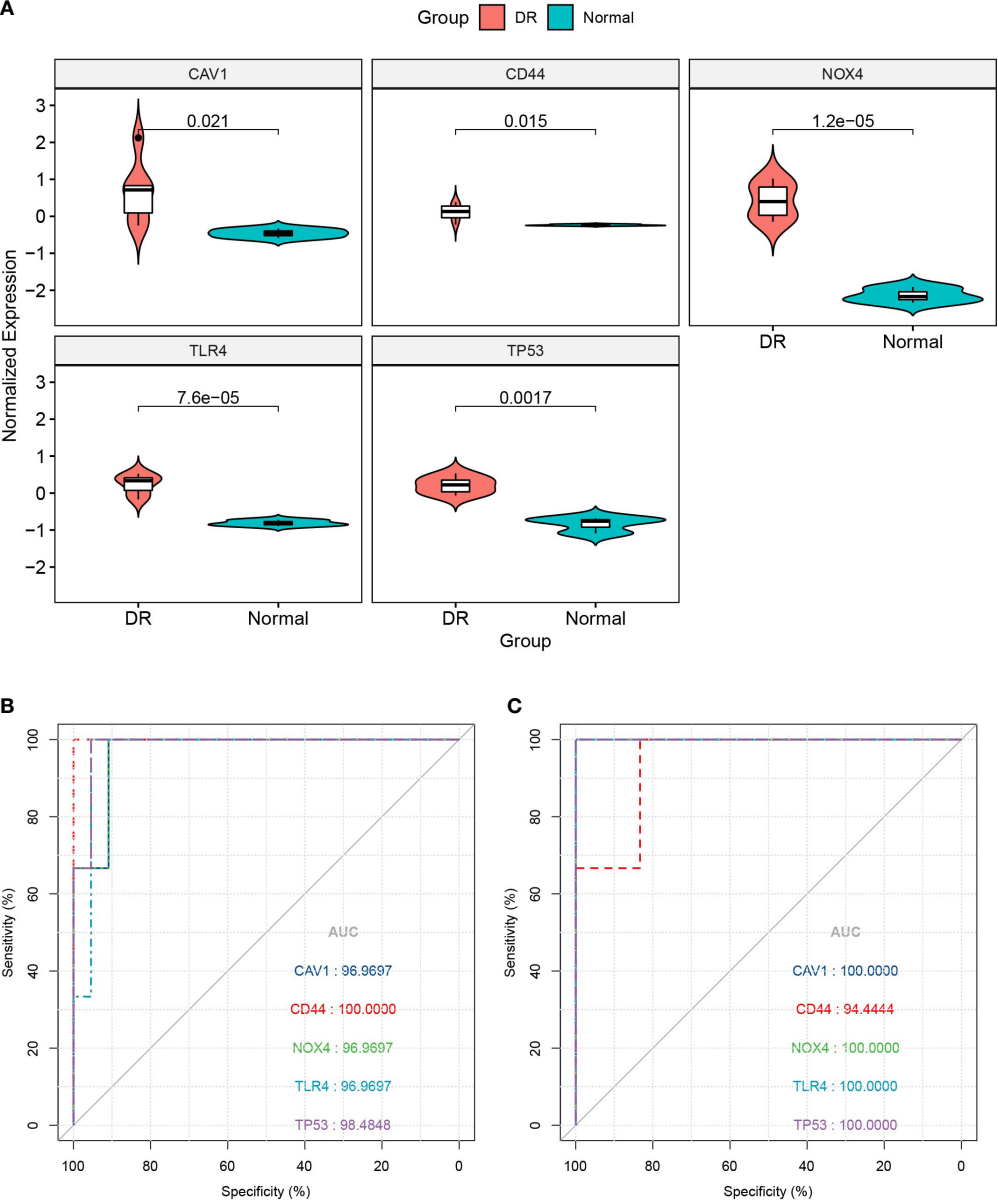
Database validation of ferroptosis-related hub genes (FRHGs). **(A)** Validation of FRHGs in the GSE60436 dataset. **(B)** ROC curve of FRHGs in the GSE102485 dataset. **(C)** ROC curve of FRHGs in the GSE60436 dataset. DR, diabetic retinopathy.

We utilized the GSE60436 dataset to validate the five FRHGs. The results revealed that the expression of these genes was higher in the DR samples than in the normal samples (all *p* < 0.05) (**Figure 5A**), which is consistent with the results of the GSE102485 dataset. Subsequently, ROC curve monofactor analysis was performed. The results revealed that the diagnostic accuracies of *CAV1*, *CD44*, *NOX4*, *TLR4*, and *TP53* for DR were 96.97%, 100.00%, 96.97%, 96.97%, and 98.48% in the GSE102485 dataset, respectively (**Figure 5B**), and 100.00%, 94.44%, 100.00%, 100.00%, and 100.00% in the GSE60436 dataset, respectively (**Figure 5C**). These results showed that the five FRHGs have great diagnostic potential for DR.

### 3.6 Immune infiltration analyses

**Figure 6:**
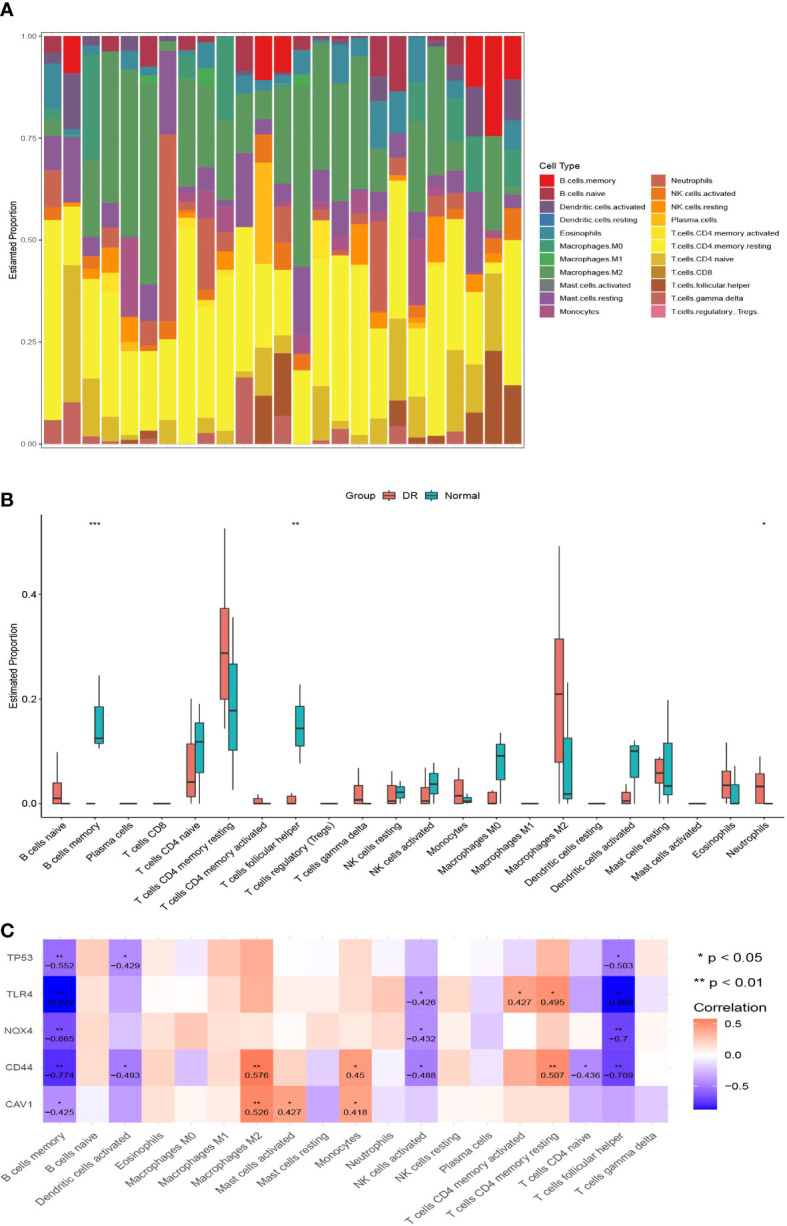
Immune infiltration analyses. **(A)** The histograms of 22 immune cell proportions in DR samples and normal samples. **(B)** The box plot of differences in immune infiltration in the two groups. *p < 0.05, **p < 0.01, ***p < 0.001. **(C)** The correlation between ferroptosis-related hub gene expression and different immune cells; the numbers in the cell represent the correlation coefficient. DR, diabetic retinopathy.

We also performed CIBERSORT immune cell infiltration analyses. The histograms in **Figure 6A** show the proportions of 22 infiltrating immune cells in each sample. There were some differences in immune infiltration between the DR and normal samples. Specifically, the DR samples had a lower memory B cell ratio, lower T follicular helper cell ratio, and higher neutrophil ratio than in the normal samples (**Figure 6B**). Regarding the correlation between FRHG expression and immune cell infiltration, the expression of *CD44*, *NOX4*, *TLR4*, and *TP53* showed a significantly negative correlation with the proportion of memory B cells and T follicular helper cells, and the expression of *CAV1* showed a significantly negative correlation with the proportion of memory B cells (**Figure 6C**).

### 3.7 Molecular docking

**Figure 7:**
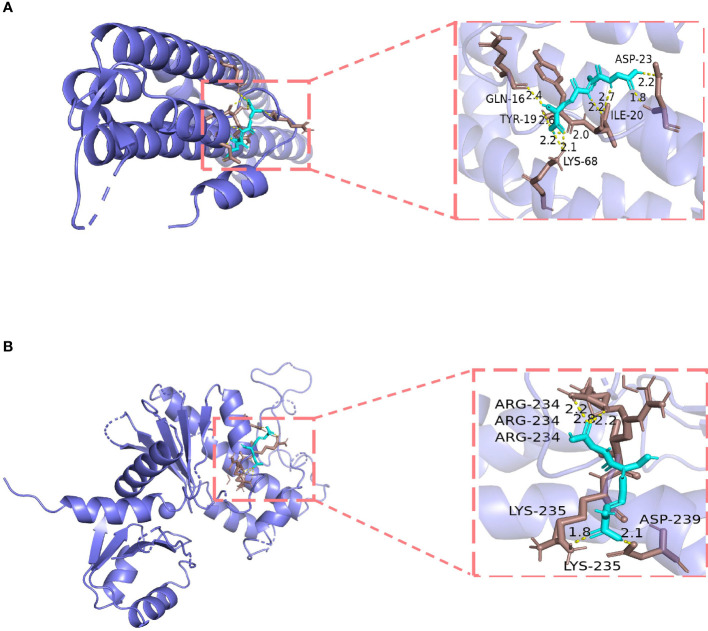
Molecular docking models of glutathione (GSH) binding to its targets. **(A)** GSH binds to CAV1. **(B)** GSH binds to TLR4.

By searching various databases, CAV1, NOX4, and TLR4 were confirmed as potential targets of GSH. Because the protein structure of NOX4 was not found in the PDB database, we only investigated the possibility of direct binding of GSH to CAV1 and TLR4 by molecular docking. The results showed that GSH possessed high binding affinity for CAV1 (PDB ID: 5IJP) (27) and TLR4 (PDB ID: 5JIC) (28). The results demonstrated that GSH forms hydrogen bonds with amino acid residues Gln-16, Asp-23, Tyr-19, Ile-20, and Lys-68 of CAV1; the free binding energy was −3.74 kcal/mol. For TLR4, GSH forms hydrogen bonds with Arg-234, Lys-235, and Asp-239; the free binding energy was −5.65 kcal/mol. The details of the binding affinities are shown in **Figures 7A, B**.

### 3.8 External validation of GPX4, SLC7A11 and FRHGs

**Figure 8:**
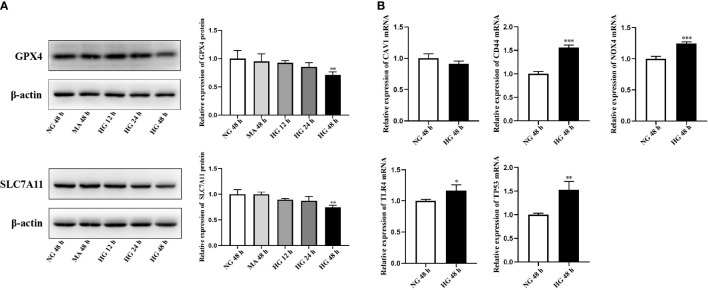
External validation of GPX4, SLC7A11 and ferroptosis-related hub genes (FRHGs). **(A)** The protein levels of GPX4 and SLC7A11 were evaluated in cell samples by western blot. **(B)** The mRNA levels of CAV1, CD44, NOX4, TLR4, and TP53 were evaluated in cell samples by qRT-PCR. NG, normal control group; MA, Mannitol; HG, high-glucose group. *p < 0.05 vs NG 48 h, **p < 0.01 vs NG 48 h, ***p < 0.001 vs NG 48 h.

HRCECs were cultured in the medium containing 30 mmol/L glucose to simulate the DR model *in vitro*. Firstly, we analyzed the differential expression of GPX4 and SCL7A11, the markers of ferroptosis, among NG group, MA group and HG group using western blot. The results showed that the protein levels of GPX4 and SLC7A11 were significantly downregulated in HRCECs under the high-glucose environment for 48 h (**Figure 8A**). The FRHGs were then validated using qRT-PCR analysis. The results showed that the expression of *CD44*, *NOX4*, *TLR4*, and *TP53* was significantly upregulated in HRCECs under the high-glucose environment for 48 h compared with the low-glucose environment, which was consistent with that of bioinformatics analysis (**Figure 8B**).

## 4 Discussion

DR, characterized by ischemic microvascular disease of the retina and retinal neurodegeneration, is a highly specific complication of diabetes with complex multifactorial pathophysiology, which finally leads to visual impairment or blindness (29). Emerging evidence from a series of *in vivo* and *in vitro* studies revealed that ferroptosis, a new type of iron-dependent programmed cell death linking metabolism, disease, immune cells, and targeted therapy, is closely associated with the pathophysiological states of various ocular diseases, such as corneal alkali burn, glaucoma, age-related macular degeneration, and retinitis pigmentosa (6, 30–32). Furthermore, targeting ferroptosis is a promising treatment for ocular diseases (6, 31). Some studies indicated that the inhibition of ferroptosis can alleviate cell death more effectively than the inhibition of apoptosis and necrosis in age-related macular degeneration (9). Most recently, a growing number of studies indicate that ferroptosis may be closely involved in the development and progression of DR (6). Ferroptosis is involved in oxidative stress-induced RPE cell and HRCEC death under high-glucose conditions. An *in vitro* study showed that glia maturation factor beta (GMFB), upregulated in the vitreous at a very early stage of diabetes, can induce ferroptosis in RPE cells by impairing lysosomal acidification and ultimately damaging retinal function (33). Another study showed that high-glucose triggers ferroptosis in HRCECs by upregulating tripartite motif containing 46 (TRIM46) and inducing ubiquitination and accelerated clearance of GPX4 (8). However, existing reports on the underlying molecular mechanisms of ferroptosis in the field of DR are still preliminary and limited in scope. Thus, we analyzed transcriptome datasets based on bioinformatics to investigate the potential pathogenesis of iron metabolism in the occurrence and progression of DR. Clarifying the interrelationship between ferroptosis and DR may identify novel biomarkers for its diagnosis and pharmacological compounds for its targeted treatment, which could provide new ideas for treatment regimens with ferroptosis as the therapeutic target.

We identified 40 ferroptosis-related DEGs between the DR and normal samples. GO and KEGG enrichment analyses revealed that these ferroptosis-related DEGs were mainly enriched in the apoptotic signaling pathway, reactive oxygen species metabolic process, response to oxidative stress, ferroptosis, p53 signaling pathway, and lipid and atherosclerosis terms, which have been reported to be associated with DR pathogenesis (8, 34–37). Interestingly, our results highlighted the involvement of these ferroptosis-related DEGs in the intrinsic apoptotic signaling pathway, regulation of apoptotic signaling pathway, and intrinsic apoptotic signaling pathway in response to DNA damage terms, consistent with previous findings that DR is affected by crosstalk between apoptosis and ferroptosis mechanisms (38, 39). The results of enrichment analyses confirmed the validity of the ferroptosis-related DEGs identified in our study. Theoretically, it is not difficult to determine that ferroptosis contributes greatly to DR. Moreover, we further analyzed the expression of GPX4 and SCL7A11, ferroptosis-related markers, was downregulated in HRCECs under the high-glucose environment, suggesting that ferroptosis is one of the pathologic mechanisms involved in DR. However, the ferroptosis-related genes and their associated terms and pathways found in our study have not been fully elucidated, especially p53 signaling pathway. It is reported that this pathway is involved in regulating metabolism, immune response, neurodegeneration and tissue ischemia/reperfusion injuries by promoting or inhibiting ferroptosis (40). But the specifically ferroptosis-related mechanism of p53 signaling pathway in DR has not been reported yet, and in-depth experimental investigation and discussion are required. We speculate that the occurrence and progression of DR are the result of crosstalk among multiple genes and pathways.

Through integrated bioinformatics analyses, we identified and validated five FRHGs (*CAV1*, *CD44*, *NOX4*, *TLR4*, and *TP53*) with great diagnostic potential for DR. Interestingly, previous studies have also indicated that these genes were upregulated in DR (41–45), although our qRT-PCR results showed that the expression of CAV1 was inconsistent with the results of bioinformatics analysis and previous studies. We speculated that because the differences in cell culture condition and vitality might provide different results. *CAV1* encodes a transmembrane protein that is the main component of caveolae in plasma membranes and is associated with multiple cellular functions including signal transduction, cholesterol homeostasis, and endocytosis (41). Increased CAV1 expression in the retinas of patients who are diabetic can enhance Toll-like receptor signaling and proinflammatory cytokine release, leading to a breakdown of the blood-retinal barrier (41, 46). CD44 is a receptor for extracellular matrix proteins and polysaccharides, as well as a significant regulator of neovascularization (47). Zhang et al. have shown that the interaction of CD44 with phosphorylated moesin leads to less pericyte coverage and disruption of vessel integrity, which may contribute to neovascularization in DR (48). NOX4 is a member of the NADPH oxidase family of enzymes, which catalyze the reduction of molecular oxygen to various reactive oxygen species (49). Previous studies have implicated the activation of NOX4 in DR blood-retinal barrier breakdown, retinal neovascularization, and inflammation (43, 50). TLR4 is a Toll-like receptor that plays an important role in the initiation of inflammatory and immune responses (39). Recent evidence has shown that TLR4 ligand- TLR4 binding initiates downstream signaling cascades, such as PI3K, p38/MAPK, and NF-κB, resulting in the development of inflammation, neovascularization, oxidative stress, and neurodegeneration, all of which are involved in DR pathogenesis (44, 51). TP53, activated in response to diverse stressors to regulate the expression of target genes inducing cell cycle arrest, apoptosis, senescence, and DNA repair, has recently been recognized as a metabolic regulator (52). Hyperglycemia increases the transcription and expression of TP53, whose codon 72 polymorphism is significantly associated with diabetic complications, including diabetic retinopathy, in patients with type 1 or type 2 diabetes (45, 52, 53). Based on machine learning algorithms, we established a novel reliable model, and these five FRHGs may represent a molecular signature for the diagnosis of patients with DR. Studies have revealed that these ferroptosis-related genes can regulate ferroptosis in liver fibrosis, heart failure, Alzheimer’s disease, and tumors (54–58). However, the exact molecular mechanisms involved in influencing DR *via* ferroptosis remain unclear. Thus, further exploration of their ferroptosis-related functions could provide novel research directions.

Chronic inflammation and leukocyte stasis play central roles in the pathogenesis of DR. An imbalance in iron homeostasis can also affect the function, differentiation, and death of immune cells (59). Thus, we utilized CIBERSORT to analyze the immune microenvironment to investigate the molecular immune mechanisms associated with ferroptosis in DR. The results showed significantly decreased proportions of memory B cells and T follicular helper cells and increased proportion of neutrophils in DR samples, which were consistent with previous studies (60, 61). Unsurprisingly, the high expression of *CAV1*, *CD44*, *NOX4*, *TLR4*, and *TP53* was linked to lower proportions of memory B cells and T follicular helper cells in DR. According to the above findings, we hypothesize that the five FRHGs are involved in the chronic inflammation and immune processes of DR occurrence and progression by affecting the immune microenvironment. The cooperative interactions of ferroptosis, immune responses, and inflammation in DR might be multilinked and complicated, and remain to be elucidated in future studies.

We investigated the possible use of GSH in treating DR by targeting FRHGs, as GSH depletion triggers ferroptosis (62). As expected, GSH was predicted to act on multiple targets (CAV1, NOX4, and TLR4) to produce synergistic pharmacological activities, and the binding affinities of GSH to CAV1 and TLR4 were predicted to be strong. A previous study showed that ferroptosis susceptibility was enhanced by increased ERK pathway activation due to CAV1 overexpression in human rhabdomyosarcoma cells, and antioxidant molecules such as GSH could alleviate ferroptosis (63). Another study revealed that ferrostatin-1, which could enhance intracellular GSH activity, was able to inhibit the ferroptosis-induced upregulation of TLR4 and the NLRP3 inflammasome to protect rat pulmonary artery endothelial cells (64). Taken together, we believe that GSH might be a promising therapeutic treatment for DR by targeting ferroptosis and undoubtedly deserves in-depth investigation in the future.

This ferroptosis-related gene signature and targeted molecule have not been previously reported in DR. However, our study still has some limitations. First, all results were based on publicly available data and existing research data; more biological experiments or clinical observations are needed to verify these findings. Second, the small sample size in the present study must also be considered. Moreover, the biological functions of these genes and the pharmacological activity of GSH need to be further validated in the *in vitro* and *in vivo* DR model, which will be the focus of our future study.

## 5 Conclusions

Based on bioinformatics technology, dataset cross-validation, and support from previous studies, we identified five FRHGs (*CAV1*, *CD44*, *NOX4*, *TLR4*, and *TP53*) associated with the pathogenesis and progression of DR. These genes are potential novel biomarkers for the diagnosis of DR and its targeted therapy. CAV1, NOX4, and TLR4 were predicted to be targets of GSH associated with ferroptosis in DR, which might contribute to the development of new DR therapies. Overall, our study provides new insights into the pathogenesis associated with ferroptosis, as well a theoretical basis for exploring new diagnostic indicators and therapeutic strategies in DR.

## Data availability statement

The original contributions presented in the study are publicly available. This data can be found here: GSE102485 and GSE60436 datasets downloaded from GEO database.

## Author contributions

JL: designed the study, collected and analyzed data and wrote the initial manuscript. XL: verified the analysis of data. YC, KL, and HZ: prepared figures and tables. ZY: guided design the study, revised and edited the manuscript. All authors read and approved the final manuscript.

## Funding

This work was supported by grants from the National Natural Science Foundation of China (No. 81860175 and 8226040184) and Science and Technology Innovation Base Construction - Clinical Medicine Research Centre Project (No. 20221ZDG02012) to ZY; The funders had no role in the study design, data collection, data analysis, interpretation, or writing of the report.

## Acknowledgments

The authors thank GEO for providing the data involved in this study.

## Conflict of interest

The authors declare that the research was conducted in the absence of any commercial or financial relationships that could be construed as a potential conflict of interest.

## Publisher’s note

All claims expressed in this article are solely those of the authors and do not necessarily represent those of their affiliated organizations, or those of the publisher, the editors and the reviewers. Any product that may be evaluated in this article, or claim that may be made by its manufacturer, is not guaranteed or endorsed by the publisher.

## Supplementary material

Supplementary Figure 1 Principal component analysis. DR, diabetic retinopathy.
