# Supplementary material for: Identification of potential ferroptosis-related biomarkers and a pharmacological compound in diabetic retinopathy based on machine learning and molecular docking
Source: Front Endocrinol (Lausanne). 2022 Nov 24;13:988506. doi: 10.3389/fendo.2022.988506 (PMC9729554; doi:10.3389/fendo.2022.988506)
Supplement: Supplementary file 1 [file Table_1.docx]

Supplementary Table 1. Primer sequences used in this study.

| Gene | Primer sequences |
| --- | --- |
| CAV1 | Forward: CGACCCTAAACACCTCAACGA  Reverse: GGCAGACAGCAAGCGGTAA |
| CD44 | Forward: TGGGTTCATAGAAGGGCACG  Reverse: CCTTTCTGGACATAGCGGGTG |
| NOX4 | Forward: TTGCTGTATAACCAAGGGCCA  Reverse: GAAGTTGAGGGCATTCACCAG |
| TLR4 | Forward: CAAGAACCTGGACCTGAGCTTTA  Reverse: GATTTGTCTCCACAGCCACCAG |
| TP53 | Forward: CCCTCCTCAGCATCTTATCCG  Reverse: GCACAAACACGCACCTCAAA |
| GAPDH | Forward: GGAAGCTTGTCATCAATGGAAATC  Reverse: TGATGACCCTTTTGGCTCCC |
