# Supplementary figures and images for: Identification of potential ferroptosis-related biomarkers and a pharmacological compound in diabetic retinopathy based on machine learning and molecular docking
Source: Front Endocrinol (Lausanne). 2022 Nov 24;13:988506. doi: 10.3389/fendo.2022.988506 (PMC9729554; doi:10.3389/fendo.2022.988506)

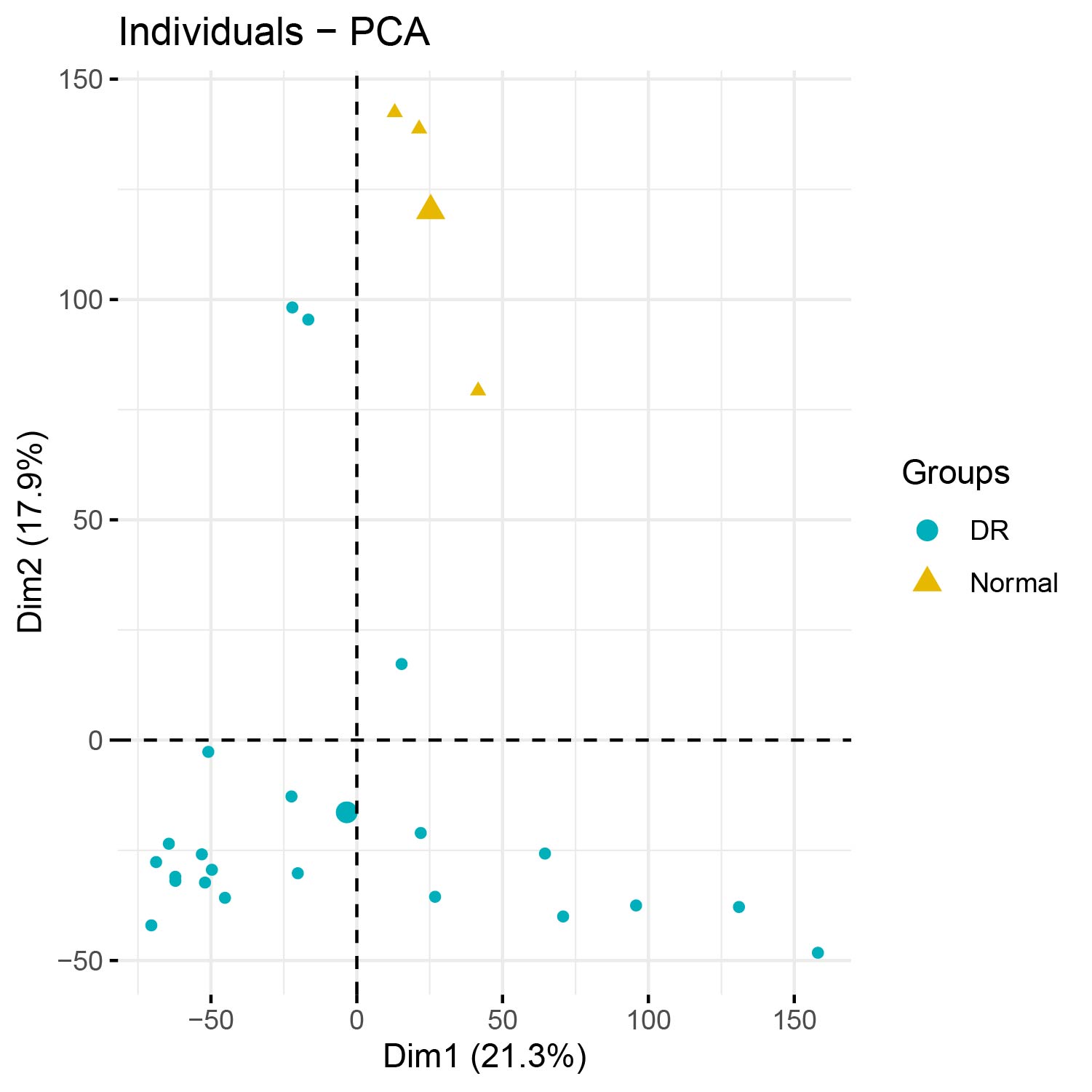

Supplement: Supplementary Figure 1 — Principal component analysis. DR, diabetic retinopathy. [file Image_1.jpeg]
